# Microvolt T-wave alternans as a predictor of mortality and severe arrhythmias in patients with left-ventricular dysfunction: a systematic review and meta-analysis

**DOI:** 10.1186/1471-2261-9-5

**Published:** 2009-01-28

**Authors:** Charlotte J van der Avoort, Kristian B Filion, Nandini Dendukuri, James M Brophy

**Affiliations:** 1 Department of Medical Technology Assessment, Radboud University Nijmegen Medical Center, Nijmegen, The Netherlands; 2 Department of Medicine, McGill University Health Center, Montreal, Quebec, Canada; 3 Department of Epidemiology, Biostatistics, and Occupational Health, McGill University, Montreal, Quebec, Canada; 4 Technology Assessment Unit, McGill University Health Center, Montreal, Quebec, Canada

## Abstract

**Background:**

Studies have demonstrated that the use of implantable cardioverter defibrillators (ICDs) is effective for the primary prevention of arrhythmic events but due to imposing costs, there remains a need to identify which patients will derive the greatest benefit. Microvolt T-wave alternans (MTWA) has been proposed to assist in this stratification.

**Methods:**

We systematically searched the literature using MEDLINE, EMBASE, Current Contents, the Cochrane Library, INAHTA, and the Web of Science to identify all primary prevention randomized controlled trials and prospective cohort studies with at least 12 months of follow-up examining MTWA as a predictor of mortality and severe arrhythmic events in patients with severe left-ventricular dysfunction. The search was limited to full-text English publications between January 1990 and May 2007. The primary outcome was a composite of mortality and severe arrhythmias. Data were synthesized using Bayesian hierarchical models.

**Results:**

We identified no trials and 8 published cohort studies involving a total of 1,946 patients, including 332 positive, 656 negative, 84 indeterminate, and 874 non-negative (which includes both positive and indeterminate tests) MTWA test results. The risk of mortality or severe arrhythmic events was higher in patients with a positive MTWA compared to a negative test (RR = 2.7, 95% credible interval (CrI) = 1.4, 6.1). Similar results were obtained when comparing non-negative MTWA to a negative test.

**Conclusion:**

A positive MTWA test predicts mortality or severe arrhythmic events in a population of individuals with severe left ventricular dysfunction. However, the wide credible interval suggests the clinical utility of this test remains incompletely defined, ranging from very modest to substantial. Additional high quality studies are required to better refine the role of MTWA in the decision making process for ICD implantation.

## Background

Implantable cardioverter defibrillators (ICDs) decrease mortality principally by detecting and treating tachyarrhythmias, the most common cause of sudden cardiac death (SCD)[1]. Although ICDs were originally used for secondary prevention among survivors of cardiac arrest or malignant arrhythmias, they have also been effective for primary prevention in high-risk patients without a history of malignant arrhythmias or SCD [2,3]. Consequently, prophylactic ICD therapy has been recommended for patients with reduced ejection fractions, resulting in a large number of potentially eligible patients [2,4,5]. Due to the substantial costs, as well as potential physical and psychological adverse effects, there is a need for better patient selection [6]. Novel diagnostic tests may have a role in improving risk stratification for ICD implantation [4].

Microvolt T-wave alternans (MTWA) represents a promising candidate test to stratify the risk among a primary prevention population [7,8]. MTWA, which usually involves an exercise treadmill test, is non-invasive and relatively inexpensive. Some studies have suggested that patients with negative MTWA tests have an extremely low risk for SCD or cardiac arrest [7] but conflicting opinions exists [9-12]. We therefore undertook a systematic review and meta-analysis to determine the utility of MTWA for risk stratification for primary prevention of patients with severe left ventricular dysfunction. Our objectives were to systematically review the existing medical literature and quantitatively summarize the utility of MTWA in performing primary prevention risk stratification.

## Methods

### Search Criteria

We performed a systematic literature search for all randomized controlled trials and prospective cohort studies published in English between January 1990, 4 years before the publication of the first clinical application of MTWA [13], and May 2007. Using the terms "MTWA" or "T wave alternans", we searched the following databases: MEDLINE, EMBASE, Current Contents, and the Web of Science, restricting our search to full text articles involving human subjects. We also hand-searched The Cochrane Library, INAHTA, and references of relevant articles, reviews, and previous meta-analyses for additional studies. Since only observational studies were identified, all elements of this meta-analysis strictly followed the guidelines described in the Meta-analysis of Observational Studies in Epidemiology (MOOSE) proposal [14].

### Inclusion Criteria

We downloaded all identified articles to Reference Manager (Version 11), and study eligibility was assessed first by examining titles and abstracts (CJA). We restricted our meta-analysis to studies examining MTWA in primary prevention. We excluded studies with patient populations involving a history of (resuscitated) cardiac death or malignant arrhythmias, Brugada Syndrome, or Long QT Syndrome. The inclusion criteria were: 1) original full length research article; 2) randomized controlled trial or prospective cohort study design; 3) exercise-induced application of MTWA; 4) human participants with left-ventricular dysfunction and no history of previous arrhythmic event; 5) reported meaningful clinical endpoints including all-cause mortality (ACM), SCD, severe arrhythmias, ventricular tachycardia (VT), ventricular fibrillation (VF), or ICD shock as a function of MTWA results. We contacted study authors to resolve important ambiguities (n = 2). Duplicate publications, studies with a follow-up of less than 12 months, and studies in patients without severe left ventricular dysfunction (due to their underlying low arrhythmic risk and fundamental differences from patients with established cardiovascular disease) were excluded. We defined patients with severe left ventricular dysfunction as those with an ejection fraction of ≤ 35%.

### Data Extraction

Two investigators (CJA and KBF) independently extracted data using a structured and pilot-tested extraction form, with discrepancies resolved by consensus. Abstracted data included: study design, study funding, inclusion and exclusion criteria, details regarding MTWA testing and classification of results, baseline demographic and clinical characteristics of participants, duration of follow-up, endpoints evaluated, multivariable analyses, and main conclusions.

We assessed study quality with a modified 7-item list based on QUADAS [15], a quality assessment tool used for diagnostic accuracy studies [see Additional file 1]. We could not use QUADAS directly as no standard reference test was available. Briefly, studies received one point for each item they reported, for a maximum score of 7. We then classified studies as having good (≥ 6), moderate (≤ 4 and < 6), or poor (< 4) quality. This quality assessment was not used to assess study inclusion or exclusion, but was included as a study characteristic.

### Statistical Analyses

Our primary analysis examined MTWA as a predictor of a composite of mortality or severe arrhythmic events in primary prevention of patients with severe left-ventricular dysfunction. In secondary analyses, we examined the use of MTWA uniquely as a predictor of mortality (all-cause mortality, cardiac death, and/or arrhythmic death). For each study, we determined: the percentage of patients tested as positive, negative, or indeterminate, and the percentage of patients who had the endpoint in each of these MTWA categories. Since some studies did not report outcomes for patients in the indeterminate category, the sensitivity and specificity reported in these studies are not valid measures. In studies with available data, we re-calculated sensitivity, specificity, and positive and negative predictive values by comparing non-negative and negative MTWA. For each MTWA category, we calculated the probability of the outcome given a positive test result (positive predictive value) and the probability of the absence of the outcome given a negative test result (negative predictive value). Given the variability in disease prevalence and duration across studies and the effect of these variables on predictive values, we decided that only the ratios comparing predictive values between categories (i.e., the relative risk) could reasonably be pooled across studies [16].

We carried out separate meta-analyses comparing each pair of MTWA categories in terms of risk ratios for each endpoint. We used a Bayesian hierarchical model with non-informative prior distributions to estimate the overall risk ratios using the WinBUGS software package [17]. We reported the posterior median and 95% credible intervals (CrI) for the parameters of interest. All plots were made using the R software package.

We also carried out two sensitivity analyses. In the first, we examined the effect of follow-up time by restricting our analysis to studies with between 1 and 2 years of follow-up. In the second, we excluded studies that included ICD shocks as part of their composite endpoint.

## Results

### Study and Participant Characteristics

**Figure 1:**
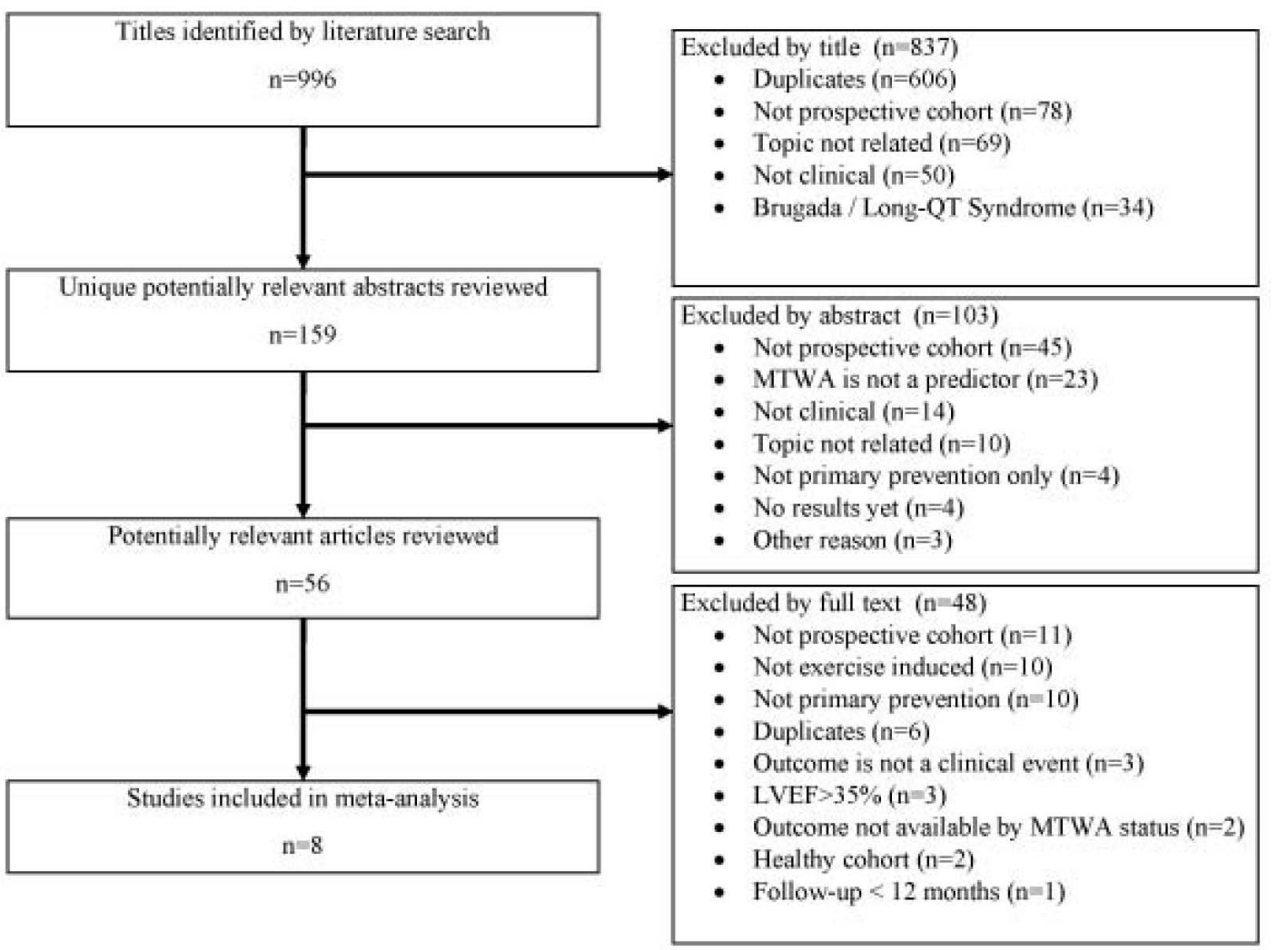
**Flow diagram of systematic literature search, study selection, and reasons for exclusion**.

No randomized controlled trials examining MTWA in primary prevention were identified. We identified 8 prospective cohort studies involving a total of 1,946 participants (Figure 1) [6,18-24]. All studies were identified in MEDLINE, although some were also found in other databases.

The characteristics of included studies are presented in Table 1. The underlying cardiac pathology varied between studies but the age and sex distributions were relatively similar. Studies also varied in sample size and duration of follow-up. Four studies were classified as being of good quality [6,18,19,22], and 4 were of moderate quality [20,21,23,24].

**Table 1:** Characteristics of prospective cohort studies examining microvolt T-wave alternans and baseline patient characteristics

**Study**	**Population**	**Sample Size**	**Men (%)**	**Age** **(Mean ± SD, years)**	**LVEF** **(mean ± SD, %)**	**Follow-Up (mean ± SD, months)**	Quality Score*
Chow 2006 [18]	ICM	768	84	67	27	18 ± 10	good
Bloomfield 2006 [6]	DCM	549	71	56 ± 10	25 ± 6	20 ± 6	good
Grimm 2003 [19]	ICD and LV > 56 mm	263	73	48 ± 12	30 ± 10	52 ± 21	good
Klingenheben 2000 [22]	CHF	107	80	56 ± 10	28 ± 7	15	good
Ikeda 2000 [20]	Post-MI	102	83	60 ± 9	NR	13 ± 6	moderate
Kitamura 2002 [21]	DCM	83^†^	81	52 ± 15	NR	21 ± 14	moderate
Sarzi Braga 2004 [24]	CHF	44^†^	89	59 ± 9	29 ± 7	19 ± 11	moderate
Sakabe 2001 [23]	DCM	30^†^	91	53 ± 16	33 ± 15	13 ± 11	moderate

Abbreviations: CHF: congestive heart failure; DCM: dilated cardiomyopathy; ICM: ischemic cardiomyopathy; ICD: implantable cardioverter defibrillator; LV: left ventricular end-diastolic volume; LVEF: left ventricular ejection fraction; MI: myocardial infarction; NR: not reported.

* Quality was assessed using an 7-item quality assessment score [see Additional file 1]. Good quality was defined as a score of 6–7, moderate quality was defined as a score of 4–5, and poor quality was defined as a score of 1–3.

**† **The sample sizes reported in the table represent the number of patients included in the analyses. A number of studies enrolled patients who were subsequently excluded from the analyses. The other numbers are based on the number of patients enrolled. These studies include Kitamura (104 patients enrolled)[21], Sarzi Braga (46 patients enrolled) [24], and Sakabe (34 patients enrolled)[23].

Differences existed in the categorization schemes of MTWA test results and in the percentage of patients in the different MTWA categories (Table 2). Of the 1,946 patients, 332 had a positive MTWA test, 656 had a negative test, 84 had an indeterminate test, and 874 were classified as non-negative. Three studies excluded indeterminate patients, preventing the calculation of valid measures of sensitivity, specificity, and positive and negative predictive values. In the remaining 5 studies, MTWA sensitivity and negative predictive values were high (Table 2). However, MTWA specificity and positive predictive values were relatively modest.

**Table 2:** Test characteristics of microvolt T-wave alternans as a predictor of mortality or severe arrhythmias

		**Patients with Endpoint (n/N)**			**Non-negative vs Negative MTWA†**			
		
**Study**	Endpoint*	**Positive** **MTWA**	**Negative** **MTWA**	**Indeterminate MTWA**	**Sensitivity**	**Specificity**	**PPV**	**NPV**
Chow 2006 [18]	ACM	78/514^††^	21/254	NR	87.6	34.8	15.2	91.7
	AD	33/514^††^	9/254	NR	78.6	33.7	6.4	96.5
Bloomfield 2006 [6]	ACM/SA/ICDS	47/360^††^	4/189	NR	92.2	37.1	13.1	97.9
Grimm 2003 [19]	CD/SA	18/137	7/72	13/54	82.6	28.9	16.2	90.3
Klingenheben 2000 [22]	CD/VT/VF	11/52	0/33	2/22	100	35.1	17.6	100
Ikeda 2000 [20]	SA	14/50	1/52	NR	NE	NE	NE	98.1
Kitamura 2002 [21]	CD	3/46	0/37	NR	NE	NE	NE	100
	SA	8/46	1/37	NR	NE	NE	NE	97.2
	CD/SA	11/46	1/37	NR	NE	NE	NE	97.2
Sarzi Braga 2004 [24]	CD	7/23	0/13	0/8	100	35.1	22.6	100
Sakabe 2001 [23]	SA	13/24	0/6	NR	NE	NE	NE	100

Abbreviations: ACM: all-cause mortality; AD: arrhythmic death; CA: cardiac arrest; CD: cardiac death; ICDS: implantable cardioverter defibrillator shock; NPV: negative predictive value; NE: not estimable; NR: not reported; PPV: positive predictive value; SA: severe arrhythmias (ventricular fibrillation or ventricular tachycardia); VF: ventricular fibrillation; VT: ventricular tachycardia;

* The inclusion of more than one clinical endpoint per row represents the use of a composite endpoint.

**† **Test characteristics are for non-negative (i.e., positive and indeterminate) vs negative MTWA. Valid estimates of the sensitivity, specificity, and PPV can only be obtained when indeterminate tests are included. Consequently, test characteristics for Grimm [19], Klingenheben [22], and Sarzi Braga [24] were recalculated using non-negative or negative MTWA. Sensitivity, specificity, and PPV for studies that excluded indeterminate tests were not estimable (NE).

**†† **The studies conducted by Chow [18] and Bloomfield [6] categorized participants as non-negative or negative MTWA. Consequently, these data represent patients with endpoints who had a non-negative test.

### Results of the Meta-Analyses

**Figure 2:**
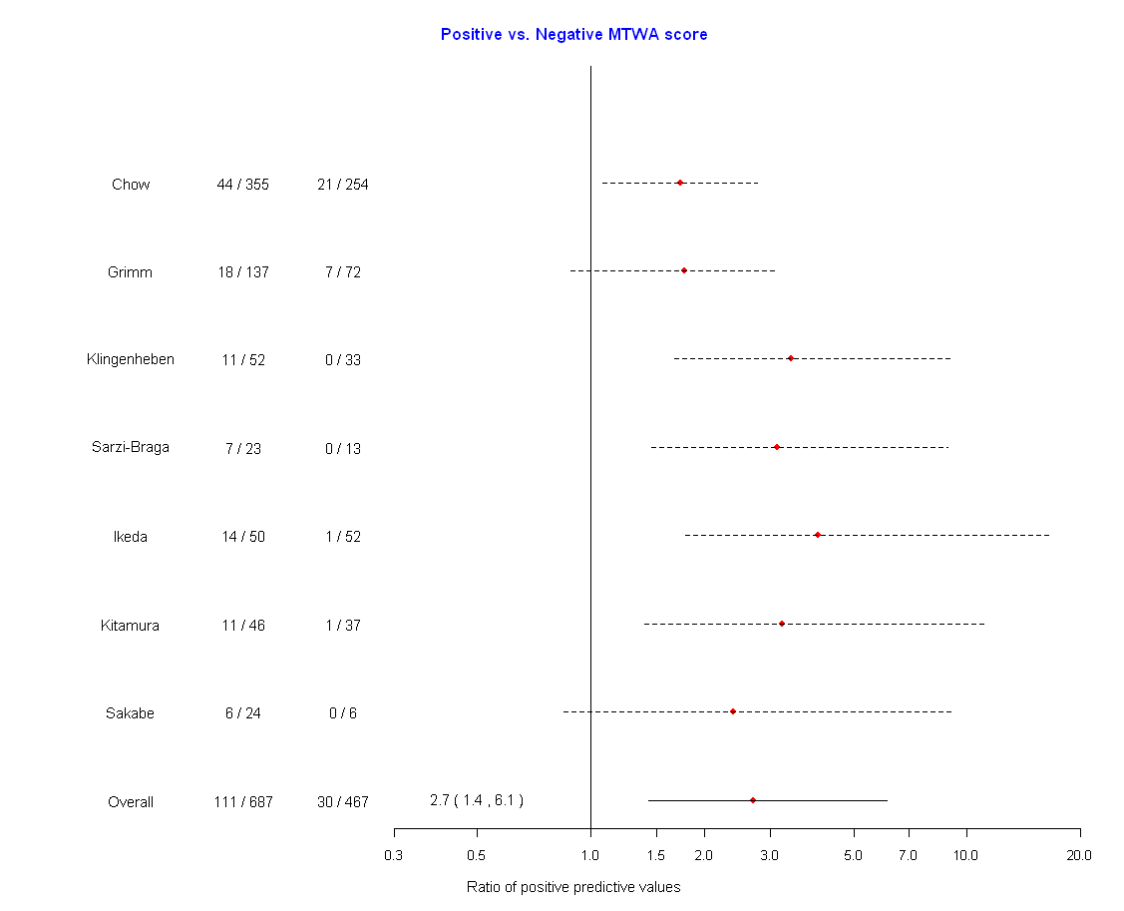
**Forest plot of the risk of mortality or severe arrhythmias among those with a positive microvolt T-wave alternans test compared with those with a negative microvolt T-wave alternans test**.

**Figure 3:**
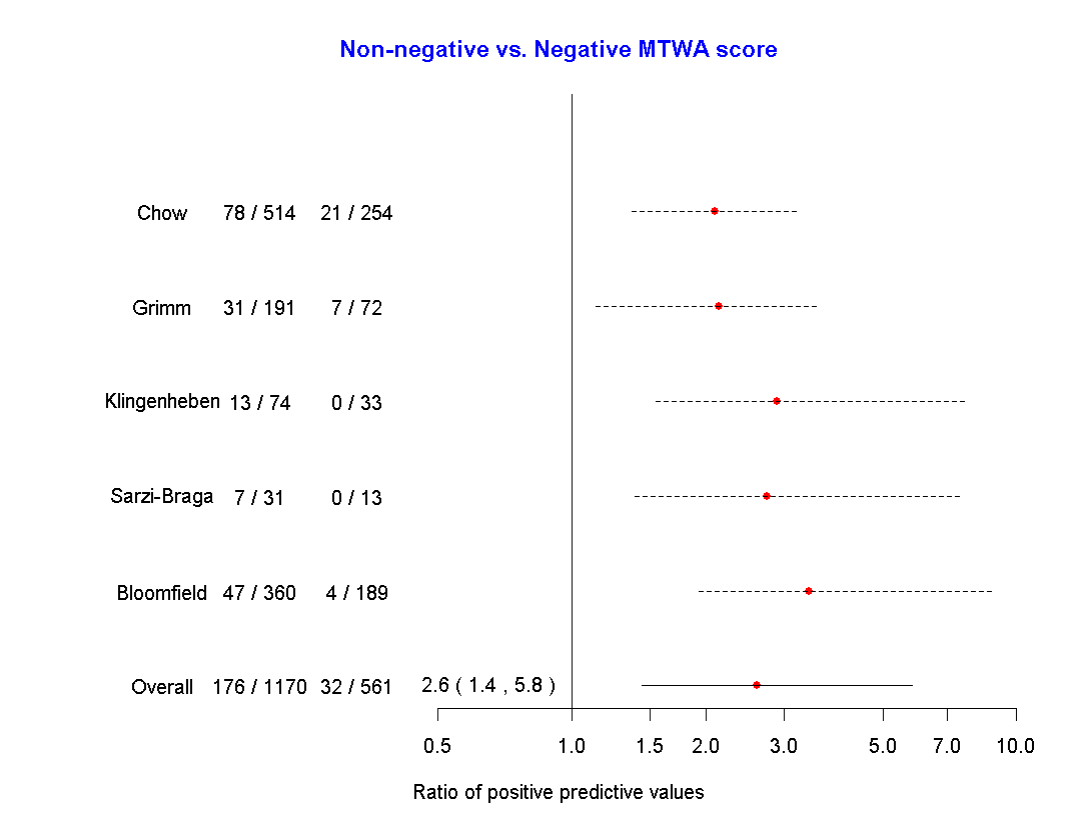
**Forest plot of the risk of mortality or severe arrhythmias among those with a non-negative microvolt T-wave alternans test compared with those with a negative microvolt T-wave alternans test**.

In our primary analysis of the composite endpoint of mortality and severe arrhythmia, we pooled data across studies to investigate the predictive ability of MTWA (Figures 2 and 3). Different numbers of studies were used in each meta-analysis, reflecting the variability in reporting of  outcomes as a function of MTWA categories. The risk of mortality or severe arrhythmic events with positive MTWA was increased compared to negative test results (RR = 2.7, 95%CrI = 1.4, 6.1) (Figure 2). Similar results were obtained when comparing non-negative to negative MTWA (RR = 2.6, 95%CrI = 1.4, 5.8) (Figure 3). There were insufficient data to make any statements regarding the predictive utility of the indeterminate group (positive vs indeterminate: RR = 1.1, 95%CrI = 0.4, 3.9; indeterminate vs negative: 2.5, 95%CrI = 0.8, 5.4). In secondary analyses, we examined MTWA as a predictor of mortality. However, with only 3 studies [18,21,24] reporting all-cause mortality, cardiac death, or arrhythmic death, this analysis provided inconclusive results (positive vs negative: RR = 1.94, 95%CrI = 0.6, 10.3; non-negative vs negative: RR = 1.94, 95%CrI = 0.4, 11.8).

We also conducted two sensitivity analyses. In the first, we restricted our analysis to the 7 studies that had follow-up durations between 1 and 2 years. In this analysis, we found that patients with a non-negative MTWA had three-times the risk of experiencing an arrhythmic event or death in the 1–2 years following MTWA testing than patients with a negative MTWA result (RR = 3.1; 95%CrI = 1.3, 9.5). These results are consistent with those obtained in our primary analysis. In the second, we excluded the study by Bloomfield and colleagues [6], which included ICD shocks as part of their composite endpoint. The inclusion of this trial appears to have had a minimal effect on our estimates (non-negative vs negative without Bloomfield: RR = 2.2, 95% CI = 1.2, 5.5).

## Discussion

This systematic review and meta-analysis examined MTWA as a predictor of mortality and severe arrhythmic events for primary prevention in patients with left-ventricular dysfunction. We were unable to identify any randomized trials investigating this diagnostic modality. Consequently, our conclusions are based on less rigorous cohort studies, which are further weakened by a lack of standardization. Nevertheless, a positive MTWA test result cumulatively predicted an increased risk of mortality and severe arrhythmic events compared with a negative test. MTWA may therefore possess some clinical utility as a stratification tool to assess short- to moderate-term risk in high-risk primary prevention populations. This is important as the majority of the economic burden associated with the prevention of arrhythmic events by ICD implantation occurs in this population [25,26]. However, the wide credible interval suggests the clinical utility may range from very modest to substantial and highlights the need for additional high-quality studies to better refine these estimates. Furthermore, our review identified a dearth of data examining the utility of MTWA for long-term risk stratification; only 1 study to date has examined the ability of MTWA to predict events after 2 years of follow-up.

MTWA as a predictor of cardiac events has been examined in several non-systematic, narrative reviews [7,8,27-31] and one systematic review. This systematic review included a meta-analysis and also concluded that MTWA predicted an increased risk of mortality and arrhythmic events [32]. Compared to the previous meta-analysis [32], which included more heterogeneous studies, we have avoided duplicate publications, added several more recent publications, and included only patient populations with significant left ventricular dysfunction. Our study is therefore more homogenous and specifically addresses the utility of MTWA in high-risk primary prevention. In addition, we have concentrated on comparing ratios of predictive values and avoided summarizing predictive values as these results are heavily dependent on study disease prevalence and follow-up time. Despite these important differences, the results of this previous study [32] are generally consistent with those of the present meta-analysis.

The utility of MTWA for predicting severe arrhythmic events in patients with left ventricular dysfunction also has been examined in 3 trials that have been recently presented at scientific meetings but not included in this meta-analysis. In the MASTER-I trial, which was presented at the Late Breaking Clinical Trial Session of the American Heart Association 2007 Scientific Sessions, 575 post-MI patients with left ventricular ejection fractions ≤ 30% and no history of severe arrhythmic events from 50 U.S. hospitals were followed for at least 2 years [33]. All patients received an ICD. The primary endpoint of the MASTER I trial was life-threatening ventricular tachyarrhythmic event, defined as either arrhythmic death or appropriate ICD discharge, and the secondary endpoints were total and cause-specific mortality. Non-negative MTWA did not appear to predict life-threatening ventricular tachyarrhythmias (hazard ratio (HR): 1.26, 95% confidence interval (CI): 0.76, 2.09), although with a wide 95% CI, we are unable to rule out a clinical difference of the same magnitude as that reported in the current meta-analysis. The MASTER I investigators did find an increase in all-cause mortality among patients with non-negative MTWA (HR: 2.04, 95%CI: 1.10, 3.78). In the MASTER II trial, the utility of MTWA was examined in 303 patients with moderate ventricular dysfunction (i.e., left ventricular ejection fraction between 31% and 40%), 48% of whom received an ICD [34]. The MASTER II trial, which excluded patients with indeterminate test results, provided inconclusive results due to a lower event rate than expected (positive MTWA vs negative MTWA: unadjusted stratified HR: 1.22, 95% CI: 0.34, 4.39; adjusted stratified HR: 1.20, 95% CI = 0.33, 4.31). In the ABCD trial, Costantini and colleagues examined the utility of MTWA and electrophysiological testing to predict different arrhythmic outcomes [35]. This study involved 566 patients with ischemic cardiomyopathy, an ejection fraction < 40%, and documented non-sustained VT, and patients were followed for 2 years. The investigators found that, at 1 year, non-negative MTWA was an important predictor of polymorphic VT, VF, and SCD (2.7% vs 0%, p = 0.04) but not of monomorphic VT (data not provided). At 2 years, non-negative MTWA was not associated with either outcome.

Although these studies provide important information regarding the utility of MTWA, we have not included them in our meta-analysis for a number of reasons. First, they have not yet undergone the rigorous peer-review associated with publication of a full manuscript. Second, in the MASTER-I trial, all patients received an ICD regardless of MTWA test. This trial therefore examines an inherently different question than the one posed in our meta-analysis. Finally, only limited information is available for each study, rending it difficult to assess the eligibility of each study relative to the inclusion/exclusion criteria of our meta-analysis. This limited information available also prevents the inclusion of these data in the analyses, particularly in the case of the ABCD trial. Despite these limitations, it is unlikely that the inclusion of these results would alter the conclusions drawn in the present study, particularly given the consistency of the point estimates obtained in these studies with our results.

Our study has a number of strengths. This is the first systematic review and meta-analysis that focuses on the use of MTWA in the setting of primary prevention in patients with left-ventricular dysfunction. By excluding studies of healthy individuals, we provide a more valid measure of MTWA specificity and negative predictive value as it pertains to clinical practice. Second, our meta-analysis included patients with indeterminate test results, avoiding a potentially important selection bias. Third, our systematic review and meta-analysis was conducted using a pre-specified protocol and in accordance with the MOOSE criteria [14]. Finally, this is the first meta-analysis to compare the different MTWA categories (i.e., positive vs negative, positive vs indeterminate, negative vs indeterminate), rather than simply grouping test results as negative and non-negative. Furthermore, our comparison of negative and non-negative MTWA was restricted to studies that reported indeterminate tests results, resulting in a more valid estimate of the effect of MTWA.

Our meta-analysis also has some potential limitations. First, we restricted our search to full-text studies published in English and thus may be affected by language and/or publication bias. Although our decision to not include studies recently published as abstracts may affect the precision of our estimates, it is unlikely to bias our results. These 3 studies have not been published because they are recently completed; the publication status is not due to the positive or negative nature of their results. Second, despite recommendations from the manufacturer strongly encouraging retesting following an indeterminate result [36], none of the studies identified in our systematic review conducted retesting. Retesting could decrease the number of patients with indeterminate test results and could potentially alter the sensitivity and specificity of MTWA. Thus, we may possibly be underestimating the potential benefits of MTWA testing. Third, there was some heterogeneity present in the individual studies included in our systematic review and meta-analysis. Studies varied in patient characteristics, duration of follow-up, study endpoints, and distribution of MTWA categories. Heterogeneity was also present in test classification, with some studies grouping patients into 3 categories (positive, negative, and indeterminate), some grouping patients into negative and non-negative, and the remaining studies excluding indeterminate tests. However, our results, although inconclusive due to wide CrIs that include both no effect and clinical important differences, suggest that there is no difference in risk of mortality or severe arrhythmic events between patients with positive MTWA and those with indeterminate results. Consequently, grouping patients as negative and non-negative is likely a valid approach. Fourth, some studies excluded indeterminate tests when calculating sensitivities and specificities. Where possible, we re-calculated the sensitivity, specificity, and positive and negative predictive values comparing non-negative and negative MTWA. However, we were unable to re-calculate these test characteristics for all studies. We also examined MTWA using the ratio of positive predictive values, which is not affected by the underlying disease prevalence. Fifth, we were limited to aggregate data and thus were unable to compare the predictive ability of MTWA to those of other tests, including New York Heart Association Class and left ventricular ejection fraction. Sixth, the ability of MTWA to predict events occurring 2 or more years after testing remains unclear. Finally, all studies included in our meta-analysis were observational studies. The potential effects of selection bias and confounding must be considered when interpreting their results.

## Conclusion

Although the body of evidence is far from ideal, MTWA appears to predict mortality and severe arrhythmias occurring within one to two years in patients with left-ventricular dysfunction and no previous history of ventricular arrhythmias. Patients with positive or indeterminate tests are at higher risk of mortality and severe arrhythmic events than patients with negative MTWA, potentially aiding the identification of patients most likely to benefit from prophylactic ICD implantation and thereby perhaps improving the cost-effectiveness of this therapy. There remains a need to examine MTWA in well-conducted randomized controlled trials as well as the ability of MTWA to predict long-term outcomes. While awaiting further quality studies, physicians and policy makers may wish to consider MTWA to help identify patients in the greatest need of aggressive primary prevention and ICD implantation.

## Competing interests

The authors declare that they have no competing interests.

## Authors' contributions

JMB conceived of the study idea, and CJA, KBF, and JMB contributed to the study design. CJA conducted the literature review. CJA and KBF performed the data extraction and drafted the manuscript. ND designed and conducted the statistical analyses. All authors were involved in revising the article for important intellectual content, interpreting the data, and approved the final version to be published.

## Pre-publication history

The pre-publication history for this paper can be accessed here:



## Supplementary Material

Additional file 1 **Quality assessment scale.** This scale, based on QUADAS [15], was used to assess the quality of studies included in the meta-analysis. Click here for file
