# Supplementary material for: Microvolt T-wave alternans as a predictor of mortality and severe arrhythmias in patients with left-ventricular dysfunction: a systematic review and meta-analysis
Source: BMC Cardiovasc Disord. 2009 Jan 28;9:5. doi: 10.1186/1471-2261-9-5 (PMC2653469; doi:10.1186/1471-2261-9-5)
Supplement: Additional file 1 — Quality assessment scale. This scale, based on QUADAS [15], was used to assess the quality of studies included in the meta-analysis. [file 1471-2261-9-5-S1.pdf]

ADDITIONAL FILE 1 – QUALITY ASSESSMENT SCALE

Quality Assessment

Was the number of participants more than 50?	□ Yes □ No □ NA
Was the number of participants more than 100?	□ Yes □ No □ NA
Was the follow-up completed by more than 80% of the participants?	□ Yes □ No □ NA
Were reasons for withdrawal from the study explained?	□ Yes □ No □ NA
Were indeterminate test results included in the analysis?	□ Yes □ No □ NA
Were the results interpreted correctly?	□ Yes □ No □ NA
Was the study clinically relevant?	□ Yes □ No □ NA
SCORE (count YES, max. 7)	………………

Quality Classification:

Good Quality: 6-7 items answered yes
Moderate Quality: 4-5 items answered yes
Poor Quality: 1-3 items answered yes
